# Analysis of the karyotype structure in *Ricolla quadrispinosa* (Linneus, 1767): inferences about the chromosomal evolution of the tribes of Harpactorinae (Heteroptera, Reduviidae)

**DOI:** 10.3897/CompCytogen.v10i4.10392

**Published:** 2016-12-09

**Authors:** Angélica Nunes Tiepo, Larissa Forim Pezenti, Thayná Bisson Ferraz Lopes, Carlos Roberto Maximiano da Silva, Jaqueline Fernanda Dionisio, José Antônio Marin Fernandes, Renata Da Rosa

**Affiliations:** 1 Departamento de Biologia Geral, CCB, Universidade Estadual de Londrina, Rodovia Celso Garcia Cid, PR 445, Km 380, Caixa Postal 6001, CEP 86051-970, Londrina, PR, Brasil; 2 Instituto de Ciências Biológicas, Universidade Federal do Pará, Belém, Universidade Federal do Pará, 66075-110; PA, Brasil

**Keywords:** holokinetic chromosomes, speciation, DAPI, heterochromatin, reproductive isolation, chromosomal rearrangements

## Abstract

The subfamily Harpactorinae is composed of six tribes. Phylogenetic studies bring together some of Harpactorinae tribes, but by and large the data on evolutionary relationships of the subfamily are scarce. Chromosome studies are of great importance for understanding the systematics of different groups of insects. For Harpactorinae, these studies are restricted to some subfamilies and involved only conventional chromosome analysis. This work analyzed cytogenetically *Ricolla quadrispinosa* (Linneus, 1767). The chromosome number was determined as 2n = 24 + X_1_X_2_Y in males. In metaphase II the autosomal chromosomes were organized in a ring with the pseudo-trivalent of sex chromosomes in its center. After C-banding followed by staining with DAPI, AT-rich blocks in autosomes were observed and the negatively heteropycnotic sex chromosomes. The data obtained, together with existing data for other species of the group, indicated that different chromosomal rearrangements are involved in the evolution of the species. In addition, a proposal of karyotype evolution for the subfamily, based on existing phylogenetic studies for the group is presented.

## Introduction

Reduviidae are the largest family of predatory insects of the suborder Heteroptera, consisting of approximately 7000 species (Kaur et al. 2009, Weirauch et al. 2014). Harpactorinae is the largest subfamily of Reduviidae, and is composed of six tribes: Apiomerini, Diaspidiini, Ectinoderini, Harpactorini, Tegeini and Rhaphidosomatini (Schuh and Slater 1995, Zhang et al. 2015). However, some authors consider Dicrotelini as a tribe (Miller 1954, Tomokuni and Cai 2002, Weirauch et al. 2014). Phylogenetic studies suggest that the first three tribes form a separate clade from the last three tribes (Davis 1969, Coscaron and Melo 2003, Zhang and Weirauch 2014, Zhang et al. 2015).

In Harpactorinae cytogenetic studies are restricted to only three of the six tribes: Apiomerini, Dicrotelini, and Harpactorini (Table 1), showing diploid numbers ranging from 12 to 30, a predominance of 24 autosomes and several sex systems (XY, XnY) (Table 1) (Kuznetsova et al. 2011, Kaur and Kaur 2013). Probable, the cytogenetical variations result from chromosomal rearrangements in autosomes and sex chromosomes. This type of alteration is an important factor in the speciation process, since causing dramatic effects on fertility (Spirito 1998, Rieseberg 2001, Livingstone and Rieseberg 2003, Nosil et al. 2009, Macaya-Sanz et al. 2011).

**Table 1.** Cytogenetic studies in Harpactorinae.

Tribe	Species	Diploid number (♂)*	Reference
**Apiomerini**	*Apiomerus lanipes*	22A+XY	Poggio et al. 2007
	*Apiomerus crassipes*	22A+XY	Payne 1912
	*Apiomerus flaviventris*	22A+XY	Ueshima 1979
	*Apiomerus spissipes*	22A+XY	Ueshima 1979
	*Apiomerus* sp.	22A+XY	Ueshima 1979
	*Heniartes huacapistana*	22A+XY	Ueshima 1979
**Dicrotelini**	*Henricohahnia typica*	24A+X_1_X_2_X_3_Y	Kaur and Kaur 2013
**Harpactorini**	*Acholla ampliata*	24A+X_1_X_2_X_3_Y	Payne 1909
	*Acholla multispinosus*	20A+X_1_X_2_X_3_X_4_X_5_Y	Troedsson 1944
	*Arilus cristatus*	22A+X_1_X_2_X_3_Y	Payne 1909
	*Coranus fuscipennis*	24A+X_1_X_2_Y	Jande 1959
	*Coranus* sp.	24A+X_1_X_2_Y	Kaur and Kaur 2013
	*Cosmoclopius nigroannulatus*	24A+X_1_X_2_X_3_Y	Poggio et al. 2007
	*Cosmoclopius poecilus*	24A+X_1_X_2_X_3_Y	Poggio et al. 2007
	*Cydnocoris crocatus*	24A+X_1_X_2_Y	Dey and Wangdi 1988
	*Euagoras erythrocephala*	24A+X_1_X_2_Y	Kaur and Kaur 2013
	*Euagoras plagiatos*	24A+X_1_X_2_Y	Kaur and Kaur 2013
	*Fitchia spinulosa*	24A+ X_1_X_2_Y	Payne 1909
	*Harpactor fuscipes*	24A+X_1_X_2_X_3_Y	Ueshima 1979
	*Irantha armipes*	24A+X_1_X_2_X_3_Y	Kaur and Kaur 2013
	*Lophocephala guerini*	24A+X_1_X_2_Y	Satapathy and Patnaik 1989
	*Montina confusa*	12+XY	Bardella et al. 2014
	*Polididus armatissimus*	10A+XY	Banerjee 1958
	*Polididus* sp.	10A+XY	Manna and Deb-Mallick 1981
	*Pselliopus cinctus*	24A+ X_1_X_2_X_3_Y	Payne 1912
	*Repipta flavicans*	18A+XY	Bardella et al. 2014
	*Repipta taurus*	24A+ X_1_X_2_X_3_Y	Kaur et al. 2012
	*Ricolla quadrispinosa*	24+X_1_X_2_Y	Present study
	*Rhynocoris costalis*	24A+X_1_X_2_X_3_Y	Kaur and Kaur 2013
	*Rhynocoris fusicipes*	24A+ X_1_X_2_X_3_Y	Dey and Wangdi 1988
	*Rhynocoris kumarii*	24A+X_1_X_2_X_3_Y	Kaur and Kaur 2013
	*Rhynocoris marginatus*	24A+ X_1_X_2_X_3_Y	Satapathy and Patnaik 1989
	*Rhynocoris* sp.	24A+X_1_X_2_X_3_Y	Kaur and Kaur 2013
	*Rocconota annulicornis*	24A+X_1_X_2_Y	Payne 1909
	*Sinea complexa*	24A+X_1_X_2_X_3_Y	Payne 1909
	*Sinea confusa*	24A+X_1_X_2_X_3_Y	Payne 1909
	*Sinea rileyi*	24A+X_1_X_2_X_3_ X_4_X_5_Y	Payne 1912
	*Sinea spinipes*	24A+X_1_X_2_X_3_Y	Payne 1909
	*Sphedanolestes himalayensis*	24A+X_1_X_2_X_3_Y	Kaur and Kaur 2013
	*Sycanus collaris*	24A+X_1_X_2_X_3_Y	Jande 1959
	*Sycanus croceovittatus*	24A+X_1_X_2_X_3_Y	Kaur and Kaur 2013
	*Sycanus* sp.	24A+X_1_X_2_X_3_Y	Manna 1951
	*Velinus nodipes*	24A+X_1_X_2_X_3_Y	Toshioka 1936
	*Velinus annulatus*	24A+X_1_X_2_X_3_Y	Kaur and Kaur 2013
	*Vesbius purpureus*	24A+XY	Manna and Deb-Mallick 1981
	*Zelus exsanguis*	24A+XY	Payne 1909
	*Zelus* sp. close to *Zelus leucogrammus*	24A+XY	Poggio et al. 2007
	*Zelus laticornis*	24A+XY	Bardella et al. 2014

* ♂ – males; A – autosomes; XY – sex system XY

Evolutionary relationships related to karyotype changes are poorly known for Harpactorinae, and the majority of karyological reports in Harpactorinae are restricted to conventional analysis without the application of banding techniques (Cai and Tomokuni 2003). The present study analyzed cytogenetically, for the first time, *Ricolla quadrispinosa* (Linneus, 1767) (Harpactorini) in order to elucidate its karyotype structure and relate this to existing data on Harpactorinae. In addition, we presented different proposals for the phylogenetic relationships of this group based on the chromosomal data available so far.

## Material and methods

### Samples and collection sites

Fifteen male specimens of *Ricolla quadrispinosa* were collected from Iguaçu National Park - Foz do Iguaçu - Brazil - 25°37'40.67"S; 54°27'45.29"W (DDM). Each individual was identified and deposited at the Federal University of Pará (UFPA).

### Chromosome preparations and conventional staining

The gonads of the adult specimens were dissected in physiological solution for insects (7.5g NaCl, 2.38g Na_2_HPO_4_, 2.72g KH_2_PO_4_ in 1L of distilled water). The testes were treated with tap water for 3 min and ﬁxed in methanol:acetic acid (3:1) for 30 min. Chromosome preparations were performed through cellular suspension by maceration in a drop of 60% acetic acid, with each gonad previously treated with 45% acetic acid. These preparations were submitted to conventional staining with Giemsa 3% and also to chromosome banding techniques. Chromosome measurements were carried out using the computer application MicroMeasure version 3.2 (Reeves and Tear 2000).

### Chromosome banding

The distribution of heterochromatin was analyzed by Giemsa C-banding according to Sumner (1972), after treatment with 0.2M HCl for 10 min at room temperature, Ba (OH)_2_ for 1 min and 40 s at 60 °C, and 2× SSC for 1 hour at 60 °C. The AT-rich bands were detected with 4’-6-diamino-2-phenylindole (DAPI), respectively, according to Schweizer et al. (1983). The slides were stained with 2µg/mL DAPI for 30 min. Slides were mounted with a medium composed of glycerol/McIlvaine buffer (pH 7.0) 1:1, plus 2.5mM MgCl_2_. All images were acquired with a Leica DM 4500 B microscope, equipped with a DFC 300FX camera and Leica IM50 4.0 software, and optimized for best contrast and brightness with iGrafx Image software.

## Results

**Figure 1.**
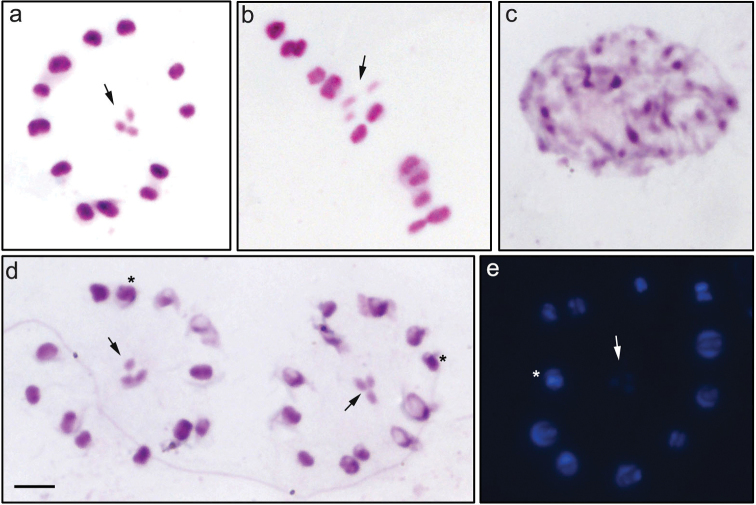
Meiocytes of *Ricolla quadrispinosa*. **A, B** conventional staining: metaphase II **C** conventional staining: interphase nucleus **D** Giemsa C-banding: metaphase II **E** DAPI staining: metaphase II. Sex chromosomes indicated by arrows. Interstitial heterochromatic block indicated by asterisk. Scale bar: 5µm.

The males of *Ricolla quadrispinosa* presented 2n = 24 + X_1_X_2_Y. In metaphase II, the autosomes are arranged in ring while the three sex chromosomes form a pseudo-trivalent in the center (Fig. 1a, b, d, e). After C-banding, sex chromosomes were shown to be negatively heteropycnotic at all stages (Fig. 1a, b). The C-banding followed by conventional staining highlighted positive heteropycnotic blocks in the interphase nuclei (Fig. 1c). It was also possible to observe positive heteropycnotic blocks in terminal regions of the majority of autosomes and in interstitial region of one pair of chromosomes (Fig. 1d).

The fluorochrome staining with DAPI performed after the C-banding revealed several AT-rich blocks in the autosomes, which were located in both the terminal and interstitial regions of the autosomes while the sex chromosomes were shown to be negatively heteropycnotic (Fig. 1e).

## Discussion

The number of autosomes observed in *Ricolla quadrispinosa* (24) was similar to that revealed in the most species of the tribe Harpactorini (Table 1), and represents the karyotype conservation regarding the number of autosomes in this group. On the contrary, the multiple sex system observed in *Ricolla quadrispinosa* (X_1_X_2_Y) has only been reported in another eight species (Table 1) (Kaur and Kaur 2013, Jande 1959, Dey and Wangdi 1988, Payne 1909, Satapathy and Patnaik 1989). Cytogenetic data exist only for three tribes: Apiomerini, Dicrotelini, and Harpactorini (Table 1). These studies are scarce considering the great diversity of the subfamily, with approximately 2800 species (Weirauch et al. 2014).

Within Harpactorinae, there is a very striking karyotype conservation in the Apiomerini tribe, where all species studied so far have presented 2n = 22 + XY (Table 1). As of now, these data support the proposed phylogeny for the group (Tomokuni and Cai 2002, Weirauch 2008, Hwang and Weirauch 2012, Zhang et al. 2015), where Apiomerini form a clade separate from Harpactorini. Analyzing the existing cytogenetic data and those obtained by us, a large karyotype variation within Harpactorini can be seen with 2n = 12 to 2n = 30 and different sex systems (Table 1), which reinforces its phylogenetic distance from Apiomerini.

According to Poggio et al. (2007), the ancestral chromosome number in Reduviidae is 2n = 28, with XY system, while the karyotypes with 22 autosomes are more common in Reduviidae (Ueshima 1979). Considering this, two evolutionary trends may be proposed for Harpactorinae: (i) reduction in the number of autosomes through episodes of chromosomic fusion, and (ii) increase in the number of sex chromosomes due to chromosomic fission events. Thus the occurrence of fissions and fusions probably gave rise to the karyotype *Ricolla quadrispinosa*, and put the Apiomerini species in a condition closer to an ancestral karyotype

In the Harpactorini, twenty-one species present 2n = 24 + X_1_X_2_X_3_Y and 9 species present 2n = 24 + X_1_X_2_Y (Table 1). Karyotypes with multiple systems with a larger number of X chromosomes are observed only in two species, *Acholla multispinosus* (De Geer, 1773) (2n = 20 + X_1_X_2_X_3_X_4_X_5_Y) and *Sinea rileyi* Montandon, 1893 (2n = 24 + X_1_X_2_X_3_X_4_X_5_Y). Although the variation in the sex chromosome systems is large, the number of autosomes is the same in the majority of species. In Heteroptera, the most common sex mechanism is XX/XY (Papeschi and Bressa 2006). Two hypotheses regarding the evolution of sex systems in Heteroptera have been proposed. The first hypothesis suggests that advanced Heteroptera, derived from the extinct group Gerromorpha, still have the plesiomorphic condition X0; thus, the XX/XY system is derived (Ueshima 1979). In contrast, the second hypothesis suggested that the X0 system is derived from the ancestral system XY (Nokkala and Nokkala 1983, 1984, Grozeva and Nokkala 1996). This last hypothesis appears to be plausible, since studies by Grozeva et al. (2014) in *Xenophyes cacus* Bergroth, 1924 (Peloridiidae, the sister group of Heteroptera) show a tendency to lose the Y chromosome during evolution. Regarding the origin of multiple sex systems, Ueshima (1979) and Papeschi and Bressa (2006) state that they are probably the result of fragmentations of the original sex chromosomes. This would likely be the origin of the multiple sex systems of *Ricolla quadrispinosa*, which have originated by breaks in the XY sex systems of ancestors.

For Dicrotelini, the only species have been cytogenetically studied, *Henricohahnia typical* Breddin, 1900 with 2n = 28 (Kaur and Kaur 2013). If consider only the diploid number, this species would be closer to the species of the Harpactorini. However, according to the phylogeny proposed by Zhang et al. (2015), the Dicrotelini form a separate clade, closer to Apiomerini than Harpactorini. In this way, due to lack of cytogenetic studies in the group, it is not possible to trace an evolutionary line within the tribe. The analysis of more species of Dicrotelini could help to elucidate this hypothesis.

**Figure 2.**
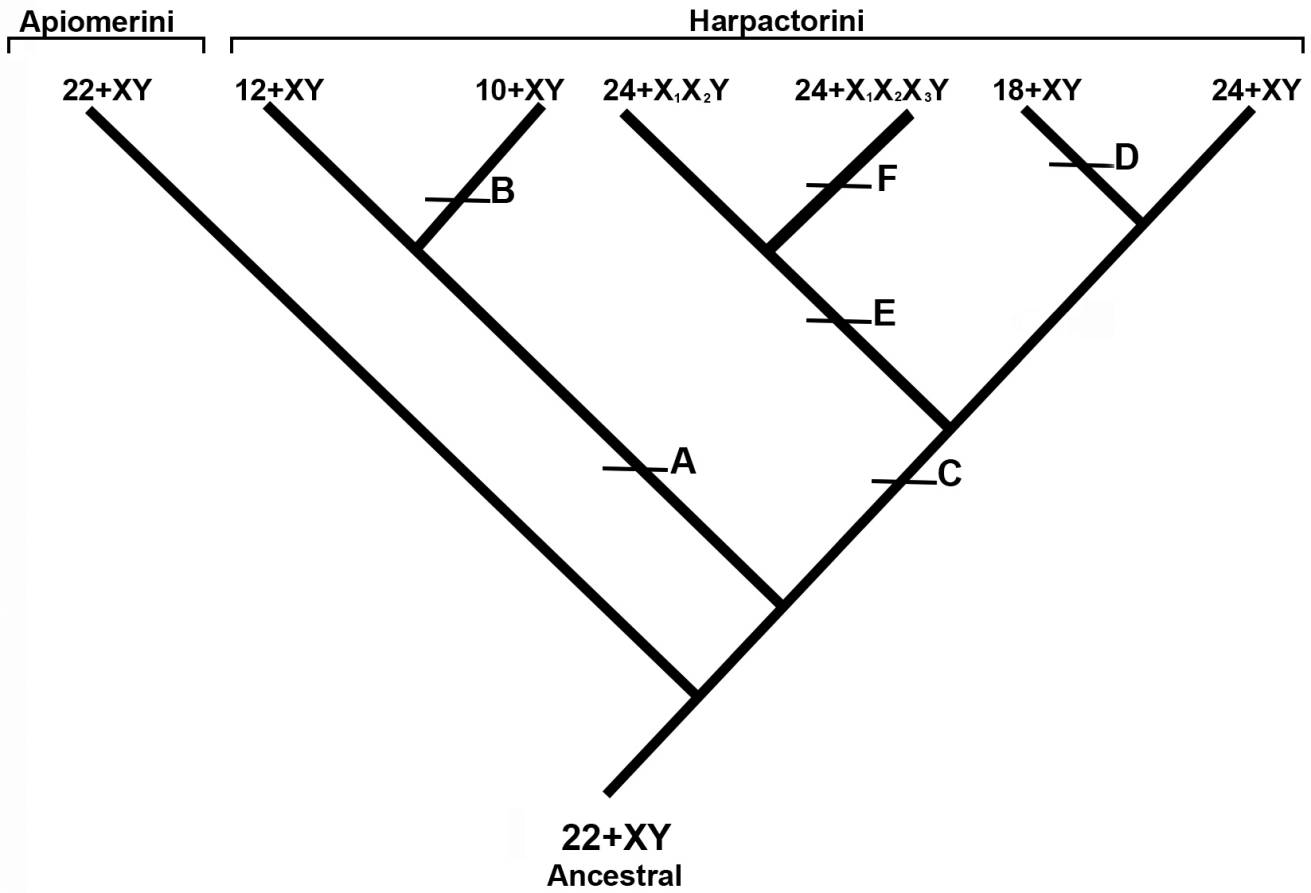
Chromosomal evolution in Apiomerini and Harpactorini. Evolutionary events marked by caps: **A** fusion of autosomes **B** fusion of autosomes **C** fission of autosomes **D** fusion of autosomes **E** fission of sex chromosomes **F** fission of sex chromosomes. Chromosomal formulae represent the diploid number. The tribe Dicrotelini was not included in scheme because only one species has been studied cytogenetically in this tribe.

Even taking into account the phylogenetic studies for the group proposed by Zhang et al. (2015), cytogenetic analyzes corroborate the differentiation of Apiomerini from Harpactorini, the former being the more conserved tribe. It is possible to group the species with similar karyotypes within Harpactorini, where those with low diploid numbers and simple sex system form separate branches from those with a higher diploid number and multiple sex systems. Considering the above, coupled with the chromosomal number found in the sister group and most of the species of the subfamilies of Reduviidae, we propose an ancestor with 2n = 24 (22 + XY) for Harpactorinae (Fig. 2). Apiomerini would have remained closer to the ancestral karyotype. Observing Figure 2, it is possible to note that an autosome fusion event (event A) would have given rise to the karyotypes of the species with 2n = 12 + XY, and then a second fusion event (event B) would have originated the karyotypes of the species with 2n = 10 + XY. These karyotypes are observed in *Montina confusa* (Stål, 1859) (Bardella et al. 2014) and two species of the genus *Polididus* Stål, 1858 (Banerjee 1958, Manna and Deb-Mallick 1981), respectively. This result can be confirmed by molecular analysis, where these genera are grouped forming a separate clade within Harpactorinae (Zhang and Weirauch 2014).

Another chromosomal alteration, the fission of autosomal chromosomes (C event) would have led to a new branch within the Harpactorini, originating 2n = 24 + XY (*Zelus* Fabricius, 1803 and *Vesbius* Stål, 1866 species) (Table 1). Also in this branch, chromosomal fusion events (D event) would originate the karyotypes with 2n = 18 + XY (Fig. 2), observed in *Repipta flavicans* (Amyot and Serville, 1843) (Bardella et al. 2014). Phylogenetically *Zelus* and *Repipta* are close (Zhang and Weirauch 2014) and occupy a separate branch within the Harpactorini distant from other species with simple sex systems, which allows us to put them in this position with respect to the karyotypic evolution. Variations in this karyotype were observed in *Repipta taurus* (Fabricius, 1803) (Kaur et al. 2012).

The multiple sex systems would have arisen by the fission of the X chromosomes of the ancestral XY system (event E) to give the karyotype 2n = 24 + X_1_X_2_Y, observed in nine species of the tribe (Table 1). A second fission event of sex chromosomes (F event) would have led to the most common karyotype observed in Harpactorini, 2n = 24 + X_1_X_2_X_3_Y, with maintenance of the number of autosomes revealed in twenty-one species of the tribe (Table 1). This explains the intermediate position of the species with these karyotypes in the phylogeny of Harpactorini (Zhang and Weirauch 2014). So far only the one species of Harpactorini, *Sinea rileyi* (Payne 1912), has a different karyotype with 2n = 24 + X_1_X_2_X_3_X_4_X_5_Y, that probably represents an isolated event of karyotypic variation, since all other species of the genus have 28 chromosomes (Table 1).

In addition to differences in the number of autosomes and sex chromosomes, in *Ricolla quadrispinosa* the sex chromosomes are presented as negatively heteropycnotic. Also in metaphase II it is possible to notice several AT rich blocks occupying the terminal and, rarely, interstitial regions of the autosomes. Different patterns of heterochromatin have been reported in other 5 Harpactorinae species (Bardella et al. 2014). Thus in *Apiomerus lanipes* (Fabricius, 1803) the presence of terminal C-DAPI^+^/CMA_3_^+^ bands in the terminal region was shown, and the heterochromatic sex chromosomes of this species exhibit different florescent patterns. In *Montina confusa* (Stål, 1859) C-DAPI^+^/CMA_3_^+^ bands were observed in both terminal regions of the two largest autosomes and sex chromosomes. *Montina confusa* also showed the third autosomal pair with a C-DAPI^+^/CMA_3_^+^ band in only one terminal region, whereas the three smaller pairs were totally C-DAPI^+^/CMA_3_^+^. In *Cosmoclopius nigroannulatus* Stål, 1860 and *Zelus laticornis* (Herrich-Schaeffer, 1853) only one of the sex chromosomes in each species was totally DAPI^+^ and CMA_3_*^+^* in each species. *Repipta flavicans* (Amyot & Serville, 1843) has not demonstrated fluorescent bands in autosomes and sex chromosomes (Bardella et al. 2014). Thus, in Harpactorinae a wide variety of different patterns of C-heterochromatin distribution between the chromosomes was revealed, that implying a large divergence in the karyotypic evolution of species of this subfamily.

Considering the influence of the chromosomal rearrangements in the speciation processes, particularly those involved in the differentiation of sex chromosomes, we can suggest that these alterations were fundamental as mechanisms of pre-zygotic reproductive isolation. It is probable that these chromosomal alterations caused the separation of groups, as different species are observed in the same geographical region, leading to a process of sympatric speciation.
